# Studying metabolic flux adaptations in cancer through integrated experimental-computational approaches

**DOI:** 10.1186/s12915-019-0669-x

**Published:** 2019-07-04

**Authors:** Shoval Lagziel, Won Dong Lee, Tomer Shlomi

**Affiliations:** 1 Faculty of Computer Science, Technion, Haifa, Israel; 2 Faculty of Biology, Technion, Haifa, Israel; 3 Lokey Center for Life Science and Engineering, Technion, Haifa, Israel

**Keywords:** Cancer metabolism, Metabolic network modeling, Metabolic flux analysis, Constraint-based modeling, COBRA, Isotope tracing, 13C-MFA

## Abstract

The study of tumorigenic rewiring of metabolic flux is at the heart of cancer metabolic research. Here, we review two widely used computational flux inference approaches: isotope tracing coupled with Metabolic Flux Analysis (13C-MFA) and COnstraint-Based Reconstruction and Analysis (COBRA). We describe the applications of these complementary modeling techniques for studying metabolic adaptations in cancer cells due to genetic mutations and the tumor microenvironment, as well as for identifying novel enzymatic targets for anti-cancer drugs. We further highlight the advantages and limitations of COBRA and 13C-MFA and the main challenges ahead.

## Inferring metabolic flux in cancer research

**Fig. 1:**
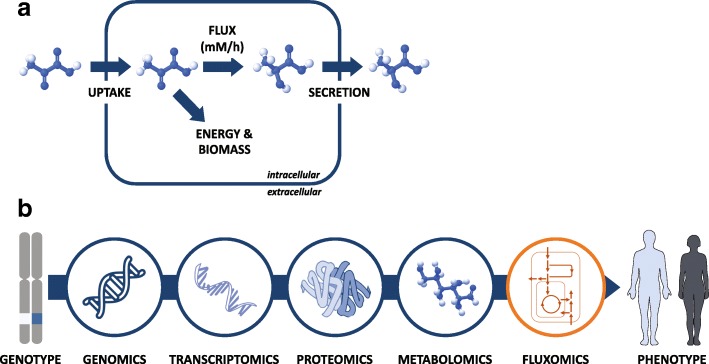
Metabolic flux describes the dynamics of cellular metabolism. **a** Metabolic nutrients are constantly consumed and metabolized to generate energy and synthesize biomass to support cell replication. **b** Metabolic fluxes provide a direct view of the cellular metabolic phenotype that is not readily evident by widely accessible ‘omics’ technologies

Cellular metabolism is a dynamic system in which metabolic nutrients are being constantly consumed and catabolized to generate energy (Fig. 1a). Proliferating cancer cells further activate anabolic pathways to produce metabolic precursors for synthesizing macromolecules, including DNA, RNA, proteins, and lipids [1, 2]. This is facilitated via a complex metabolic network consisting of thousands of biochemical reactions [3, 4]. The dynamics of metabolism can be described in terms of the rate of metabolic reactions, typically referred to as metabolic flux (denoting the rate of transformation of a substrate to product metabolites in units of moles per unit of time per cell). A major goal of cancer metabolic research is understanding how metabolic flux is rewired by tumors to support energetic and biosynthetic demands [5, 6]. Understanding tumor-specific alterations in metabolic flux facilitates the identification of induced dependency on specific enzymes whose pharmacological inhibition selectively targets cancer cells [7].

A major complication in cancer metabolic research is that, unlike the concentration of mRNA, proteins, and metabolites, metabolic flux, which reflects the cellular metabolic phenotype, is not a directly measurable quantity (Fig. 1b). However, it can be inferred through a combination of experimental and computational techniques.

The most direct approach for interrogating intracellular metabolic flux in cancer cells is isotope tracing [8–10]. This works by feeding cancer cells with isotopically labeled nutrients and measuring the isotopic labeling pattern of metabolites via mass spectrometry or nuclear magnetic resonance (NMR). We discuss here the common application of this approach in cancer cells grown in culture, though it is also utilized for in vivo studies [11, 12]. The isotopic labeling pattern of metabolites is indicative of the relative contribution of different pathways to their biosynthesis. While a manual inspection of measured metabolite isotope distributions facilitates the qualitative assessment of metabolic activities, computational interpretation via 13C-Metabolic Flux Analysis (13C-MFA) further enables quantitative inference of fluxes.

Another commonly used flux inference approach is COnstraint-Based Reconstruction and Analysis (COBRA), enabling flux assessment through genome-scale metabolic networks. COBRA has traditionally been utilized to model microbial metabolism for biotechnological and bioengineering purposes [13–15]. More recent reconstructions of genome-scale human metabolic network models enabled applying this approach for large-scale modeling of normal tissues and various human diseases, including cancer [3, 16–19]. COBRA predicts fluxes under metabolic steady state by taking into account physicochemical considerations, specifically stoichiometric mass-balance, requiring metabolite total production and consumption rates to be equal under steady state conditions. An important feature of COBRA is its ability to predict flux and metabolic rewiring by incorporating various ‘omics’ datasets, such as transcriptomics, proteomics, and metabolomics. This enables flux prediction for large collections of cell lines and tumors via existing functional genomics and metabolomics datasets, including TCGA [20], NCI60 [21], CCLE [22–24], and Connectivity Map [25].

Here, we provide a brief overview of how COBRA and 13C-MFA work (readers are referred to comprehensive reviews on COBRA [26] and 13C-MFA [27] for further technical information), recent usage of these approaches in cancer research studies, and the limitations and open challenges with each flux inference approach.

## Isotope tracing coupled with MFA

**Fig. 2:**
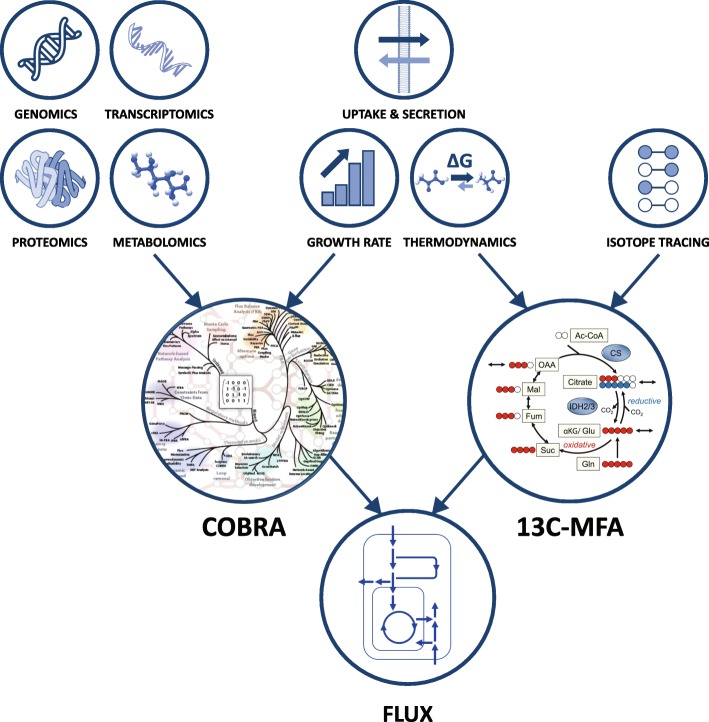
Both 13C-MFA and COBRA rely on measurements of metabolite uptake and secretion, cell biomass composition and growth rate, and information on reaction reversibility based on thermodynamic considerations. 13C-MFA further requires isotope tracing measurements and absolute concentrations of intracellular metabolites in a case of non-stationary 13C-MFA; COBRA relies on a variety of ‘omics’ datasets (genomics, transcriptomics, proteomics, and metabolomics). Inset COBRA image taken from [28]

13C-MFA calculations require a metabolic network model consisting of a set of biochemical reactions, with information on the mapping of atoms between the substrate and product metabolites (and specifically carbon atom mappings for ^13^C tracing; Fig. 2). 13C-MFA works by searching for the most plausible steady-state fluxes satisfying stoichiometric mass-balance for intracellular metabolites (i.e.*,* metabolite total production rate equals total consumption rate) for which a simulated isotopic labeling pattern of metabolites optimally matches experimental measurements [8, 27]. From an algorithmic perspective, 13C-MFA is computationally hard, requiring solving of a non-convex optimization [29]. Hence, 13C-MFA calculations are typically performed via heuristic solving of optimization problems; e.g., using Sequential Quadratic Programming (SQP) or interior-point, which do not guarantee convergence to an optimal solution. To speed up the heuristic solving, various methods were proposed to efficiently simulate metabolite isotope labeling given a possible set of fluxes [30, 31]. The most commonly used method is the Elementary Metabolite Unit (EMU) [30], implemented in a variety of user-friendly software tools, including INCA, Metran, and ^13^CFlux2 [32–34]. These tools enable straightforward inference of flux through a given metabolic network based on isotope tracing measurements. Additional measurements of metabolite uptake and byproduct secretion rates from and to media can be utilized by the above computational tools to improve 13C-MFA flux estimation. Estimates of cellular flux demands for biomass production, determined based on the macromolecular composition of cells, can be incorporated in 13C-MFA to further constrain estimated fluxes. A rigorous statistical framework enables computing flux confidence intervals, representing the extent of the uncertainty of inferred fluxes [35, 36]. Integration of measurements from multiple isotope tracing experiments is an especially useful feature of 13C-MFA that reduces the uncertainty in estimated fluxes [37].

The most common 13C-MFA approach, stationary 13C-MFA, is based on measuring metabolite labeling patterns once metabolite labeling converges to isotopic steady state. In some cases, however, this is not possible due to metabolite secretion from cells gradually changing the labeling of metabolite pools in the culture media (which in turn alters intracellular metabolite labeling) [38]. When an isotopic steady state cannot be reached, non-stationary 13C-MFA can be used to infer fluxes based on measurements of metabolite labeling kinetics [39]. Acquiring and analyzing isotope labeling kinetic data is more demanding from both experimental and computational perspectives [40]. Data analysis is performed similarly as in stationary 13C-MFA via non-convex optimizations searching for optimal fluxes, though utilizing ordinary differential equation (ODE) models to simulate metabolite isotope labeling kinetics. The simulation of metabolite isotopic labeling kinetics further requires the measurement of absolute concentrations of intracellular metabolites. In some cases, kinetic isotope tracing measurements can be directly utilized to infer flux without ODE-based simulations utilizing Kinetic Flux Profiling (KFP) [41] or cumulative isotopomer balance equations [42]. While being experimentally and computationally demanding, non-stationary 13C-MFA is advantageous in terms of being able to infer fluxes via linear pathways based on the labeling kinetics of subsequent metabolic intermediates, as compared to stationary 13C-MFA only estimating flux ratios through converging pathways producing a certain metabolite (based on the characteristic isotopic labeling pattern produced by each pathway).

13C-MFA has been frequently used for investigating cellular metabolic rewiring in response to genetic mutations in cancer, revealing the link between signaling circuitry and cancer metabolism. For example, oncogenic activations of Ras [43, 44], Akt [44], and Myc [45] were found to induce aerobic glycolysis (in accordance with the Warburg effect), glutamine consumption, and oxidation in the TCA cycle. In addition, KEAP1 mutations were shown to alter cancer redox network and oxidative pentose phosphate pathway flux [46].

Employing 13C-MFA to probe flux alterations following genetic silencing of metabolic enzymes provided means to explore enzyme importance and mechanisms: depletion of MTHFD1L, an enzyme in the mitochondrial folate cycle that produces formate, was shown to repress mitochondrial one-carbon metabolism and lead to reduced cancer invasion [47]. Deletion of Hexokinase 2 in hepatocellular carcinoma inhibits glycolysis and induces oxidative phosphorylation flux [48]. PDH deletion in lung cancer cells induces scavenging of extracellular lipids and lipogenesis through increased reductive IDH1 flux [49]. Flux rewiring due to compromised metabolite transporters was also investigated using 13C-MFA: the depletion of the mitochondrial pyruvate carrier (MPC) increased the oxidation of fatty acids and glutaminolytic flux [50]; and ablation of mitochondrial citrate transport protein (CTP) increased glucose-dependent anaplerotic flux and cytosolic reductive carboxylation for lipogenesis [51]. This suggests novel therapeutic targets, inhibiting cancer cell-specific utilization of the above nutrients or enzymes.

Metabolic rewiring due to non-genetic factors such as the tumor microenvironment has also been investigated utilizing 13C-MFA. Hypoxia promotes tumor cell reliance on reductive glutamine metabolism for lipogenesis [52, 53] and malic enzyme for NADPH production [54]. Increased reductive glutamine flux also promotes anchorage-independent growth [55]. 13C-MFA was recently employed to examine how metabolic flux in tumors differs between in vitro and in vivo conditions; e.g., human NSCLCs were shown to depend on increased PC and PDH flux and rely extensively on lactate catabolism in vivo [56, 57].

Identifying and characterizing metabolic rewiring with 13C-MFA in specific cancer cells not only contributes to our understanding of metabolic regulation but can also lead to the discovery of novel targets for anticancer drugs. For example, applied to studying the effect of PHGDH amplification in breast cancer cells, 13C-MFA revealed that de novo serine biosynthesis is responsible for up to half of the total anaplerotic flux of glutamine into the TCA cycle, suggesting that targeting the serine synthesis pathway may be therapeutically valuable in breast cancers with elevated PHGDH expression [58]. Likewise, 13C-MFA identified induced essentiality of oxidative mitochondrial metabolism in IDH1-mutant cells that can be therapeutically exploited [59].

**Fig. 3:**
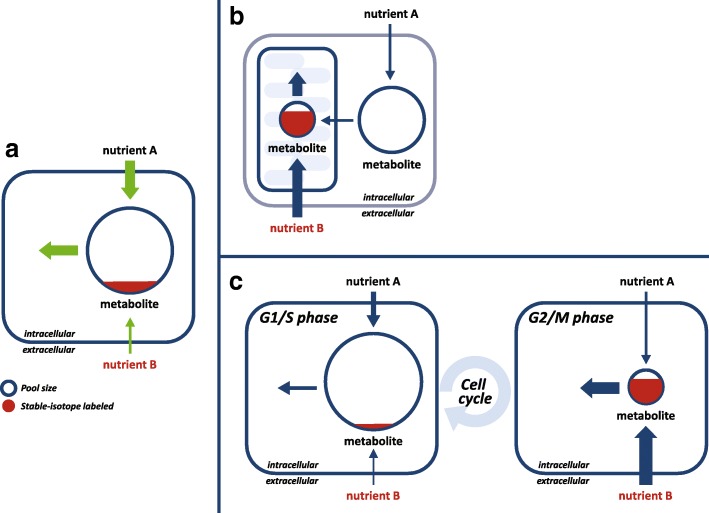
Spatial and temporal compartmentalization of cellular metabolism may bias the estimation of whole-cell level fluxes. **a** Consider the case of a metabolite synthesized from two nutrients in media: *A* and *B*. Let us assume that feeding the cells with an isotopic form of *B* leads to an isotopic steady-state in which a small fraction of the intracellular metabolite pool is labeled. In this case, 13C-MFA would infer that the relative contribution of nutrient *B* to producing the metabolite is smaller than that of *A*. However, this might not be the case when considering spatial (**b**) and temporal (**c**) compartmentalization of metabolic activities. **b** Consider the case where the metabolite is synthesized mostly from nutrient *B* in mitochondria and at a lower rate from nutrient *A* in the cytosol. If the metabolite pool size is markedly larger in the cytosol, feeding cells with labeled nutrient *B* would lead to a small fraction of the whole-cell total metabolite pool to be isotopically labeled. **c** Consider the case where in a certain cell cycle phase (e.g., G2/M) the metabolite is rapidly synthesized and mostly from nutrient *B*, while in other phases (G1/S) it is slowly produced and mostly from *A. now*, if the metabolite pool size is markedly larger in G1/S, feeding a population of cells (homogenous in terms of cell cycle phase) with labeled nutrient B would lead to a small fraction of the total metabolite pool to be labeled

A major limitation of flux inference via isotope tracing coupled with 13C-MFA regards the inference of metabolic flux in specific organelles (Fig. 3a, b). Subcellular compartmentalization is a defining characteristic of eukaryotic cells, with metabolic enzymes being localized and operating in specific organelles. For example, mitochondrial metabolism is highly inter-linked to cytosolic metabolism via the shuttling of energy and redox equivalents through the mitochondrial membrane. Furthermore, numerous isozymes catalyze the same metabolic transformation in both compartments, in some cases utilizing distinct energy and redox cofactors. Considering that mass spectrometry approaches typically measure the average whole-cell level metabolite concentrations and isotopic labeling, 13C-MFA methods are generally limited to inferring whole-cell level fluxes. Notably, not accounting for distinct metabolite isotopic labeling patterns and concentrations in different cell compartments can bias the interpretation of isotope tracing experiments and result in a false estimate of metabolic flux. This can be partially overcome by considering a metabolic network model in which metabolite pools and reactions are localized in different compartments, and inferring the isotope labeling of metabolites in specific subcellular compartments based on specific metabolite markers known to be synthesized in a specific compartment. For example, fatty acid labeling can be measured to infer cytosolic acetyl-CoA, considering that this biosynthetic activity takes place in the cytosol [60]. Mass spectrometry-based measurement of metabolic byproducts secreted to media provides information on the isotopic labeling of cytosolic metabolite pools [61]. In some cases, compartment-specific enzymes were engineered to produce reporter metabolites to infer mitochondrial and cytosolic NADPH labeling [54, 62]. Gene expression measurements and in vitro enzymatic assays were performed in specific cell lines to determine that some metabolic transformations occur solely in one compartment [61]. The expression level of alanine aminotransferase isozymes in breast cancer cell lines indicated that only the mitochondrial isoform is active, suggesting that the whole-cell level isotopic labeling pattern of alanine reflects mitochondrial pyruvate labeling [63]. To address the challenge of inferring compartment-specific metabolic flux, isotope tracing has been applied to isolated mitochondria [55, 64, 65]. However, isolation and purification of mitochondria typically involve a lengthy and perturbative process, potentially resulting in non-physiological conditions. More recently, a method was suggested to infer mitochondrial and cytosolic fluxes by rapidly fractionating isotopically labeled cells in a manner of seconds. This is shown to enable flux inference through isozymes catalyzing the same metabolic transformation in mitochondria and cytosol, and even between distinct isozymes within mitochondria, based on co-factor specificity [66].

Metabolic activities are not only spatially compartmentalized within cells but also vary with time (Fig. 3c). For example, as cells progress through different cell cycle phases, their metabolism adapts to the changing metabolic and energetic demands. Temporal compartmentalization is typically not accounted for by 13C-MFA studies relying on isotope tracing experiments performed on a population of cells that are heterogeneous in their cell cycle stage. Instead, 13C-MFA typically estimates the “average” flux through the cell population. Recently, a temporal-fluxomics method was developed for inferring metabolic flux dynamics throughout the cell cycle by performing isotope tracing experiments on a growth-synchronized population of cells [67]. This involved computational modeling of single-cell level metabolite isotopic labeling dynamics throughout the cell cycle as well as non-stationary 13C-MFA techniques. This study presented, for the first time, metabolic flux dynamics throughout the cell cycle in the central energy metabolism of proliferating cancer cells.

## Genome-scale metabolic network modeling in cancer with COBRA

COBRA predicts metabolic fluxes by considering physicochemical constraints, including stoichiometric mass-balance of intracellular metabolites, reaction reversibility based on thermodynamic considerations, and bounds on nutrient consumption and byproduct secretion rates (Fig. 2). Nutrient consumption and byproduct secretion rates in cells grown in culture are readily measurable via mass spectrometry-based analysis of metabolite accumulation and depletion from the growth media [68]. These measurements can be directly incorporated with COBRA to facilitate flux prediction. Another useful constraint is on the production rate of biomass constituents needed for synthesizing DNA, RNA, proteins, and fatty acids required to support experimentally observed cell doubling time (typically incorporated in the model via a pseudo cell-growth reaction) [69].

The high level of redundancy in the metabolic network in terms of alternative pathways typically prevents the inference of a unique set of fluxes. This is typically addressed by exploring the flux solution space via methods such as flux variability analysis [70, 71], flux coupling analysis [72], or flux sampling [73]. Alternatively, assumptions of metabolic efficiency can reduce the space of possible fluxes and predict likely metabolic phenotypes. For example, Flux-Balance Analysis (FBA) assumes biomass production with a high yield [74]; or parsimonious FBA, assuming a minimization of total fluxes needed to realize a certain metabolic objective [75]. The identification of such optimized fluxes is typically performed via efficient linear or quadratic programming algorithms. The COBRA Toolbox is a widely used MATLAB software package implementing many of the methods described in this review and others [76].

## COBRA modeling of hallmark metabolic adaptations in cancer cells via measured nutrient and uptake secretion rates

Several studies have utilized COBRA to explore the production and consumption of central energy (ATP) and redox cofactors (NAD(P)^+^/NAD(P)H). Metabolite uptake and secretion rates across the NCI-60 cancer cell lines collection were used to model fluxes in these cells, exploring different metabolic strategies used by cells to generate energy and redox cofactors and explaining the abilities of different cell lines to support respiration [77]. An analysis of fluxes in NCI-60 using uptake and secretion rates, cell proliferation rates, and DNA content showed an important contribution of one-carbon metabolism to NADPH and ATP biosynthesis [78]. The potential importance of serine and glycine metabolism to ATP production was further noted based on a molecular crowding effect in mitochondria—i.e., a limit on the total mitochondrial enzyme content per cell volume [79]. Fan et al. [80] demonstrated the importance of the cytosolic one-carbon metabolic pathway as an efficient way of producing NADPH, providing biochemical and genetic evidence for the role of this pathway in NADPH production.

Several studies used COBRA to explore overflow metabolism in cancer—i.e., excess consumption and non-efficient utilization of metabolic nutrients, including for glucose [81], glutamine [82], and serine [47]. Induced glucose consumption and fermentation into lactate under the presence of oxygen by cancer cells is known as the Warburg effect [83, 84]. This phenomenon is counter-intuitive as it provides a markedly lower ATP yield per molecule of glucose than through complete oxidation in mitochondria coupled with oxidative phosphorylation. However, utilizing FBA and considering the effect of molecular crowding (also referred to as the effect of solvent capacity), it was shown that switching to aerobic glycolysis, although of a low ATP yield, enables induced biomass production to support an increased proliferation rate [85] (as also shown by [86] using a tailored mechanistic model). In a recent study, overflow metabolism of glucose, glutamine, and serine were investigated via flux analysis of NCI-60 cell lines (utilizing measured metabolite uptake and secretion rates) [87]. This study shows that overflow glucose and glutamine metabolism is due to a constraint on the maximal catabolic capacity of mitochondria, providing excess redox and energy production that facilitates resistance to metabolic stress.

## Construction of cell line-specific metabolic models via omics data predicts metabolic gene essentiality

While measured metabolite uptake and secretion rates in a given cell line provide readily usable constraints for flux analysis by COBRA, utilizing abundant transcriptomic, proteomics, and metabolomics datasets (available for large collections of cell lines) as input for flux prediction is highly challenging. This is due to metabolic flux being regulated at multiple levels and depending on the concentration of the active enzyme (which is affected by multiple post-translational modifications), the concentration of reactants and allosteric regulators, and complex enzyme kinetic mechanisms (requiring knowledge of kinetics constants that are rarely known under physiological cellular conditions). Numerous computational techniques have been proposed to generate metabolic network models for specific tumors (i.e., context-specific models). Specifically, these methods aim to identify a subset of enzymes from a genome-scale metabolic network that is expected to be active based on the mRNA, protein, and metabolite concentrations, enzyme-specific biochemical or genetic measurements, and known cell line-specific metabolic functions. Various methods such as GIMME [88], iMAT [89, 90], MBA [91], mCADRE [92], INIT [93], PRIME [94], and FASTCORE [95] differ in terms of the specific criteria used to select the relevant set of enzymes per cell line (see review and comparison in [96, 97]).

Predictions of cell line-specific gene essentiality derived with cell line-specific metabolic network models were shown to correlate significantly with measured growth response to CRISPR-based gene knockouts [98], achieving a stronger correlation than that expected by chance or obtained for predictions made with a generic genome-scale metabolic network model. However, while various methods for predicting the effects of gene knockouts in cell lines were comprehensively compared to one another [97], the actual predictive performance of most of these methods remain somewhat unclear as information on the correlation between model predictions and measured growth inhibition effect (or sensitivity and specificity) is typically not available.

While predicting cell line-specific response to genetic silencing or chemical inhibition is technically difficult, identifying enzymes whose inhibition selectively affects cancer cells while sparing normal cells is even more challenging. This was previously addressed by searching for enzymes whose inhibition would prevent cell proliferation, while not affecting basic metabolic functionality such as ATP production [99]. Additional studies generated cell line-specific metabolic models for normal and cancer tissues, identifying cancer liabilities and predicting the response for drug inhibition of metabolic enzymes [92, 94, 100]. Yizhak et al. suggested an algorithm, Metabolic Transformation Algorithm (MTA), for identifying metabolic genes whose perturbation has a tumorigenic effect [101]; searching for genes whose change in expression in tumors is predicted to drive metabolic adaptations consistent with observed alterations in gene expression patterns. This was used to uncover FUT9 as a metabolic driver of colorectal cancer, which was validated in vitro and in mouse xenografts [102].

Another appealing approach for identifying selective anti-cancer metabolic targets is based on the concept of synthetic lethality [103]. Specifically, two genes are considered to be synthetically lethal if the perturbation of each of them separately has no effect on cell viability while their combined perturbation is lethal. In cancer cells, somatic inactivation of one gene makes its synthetic lethal partner an attractive target for selective eradication of cancer cells. This concept was used to predict synthetic lethal partners of the known metabolic tumor suppressors fumarate hydratase (FH) and succinate dehydrogenase (SDH). It successfully identified heme oxygenase (HMOX) as a synthetic lethal partner of FH, as was validated in HLRCC cells with a loss-of-function mutation in FH [104], and pyruvate carboxylase (PC) as a synthetic lethal partner of SDH, which was also later experimentally validated [105]. An extended framework was proposed for finding sets of synthetic lethal genes such that the combined knock out of which blocks a desired metabolic task, utilizing the concept of minimal cut sets [106]. A related concept of synthetic dosage lethality (SDL) represents the case where increased expression of one gene is indicative of induced dependency on another. A COBRA method developed for identifying dosage lethality effects (IDLE) revealed that the expression pattern of SDL genes is predictive of tumor size and patient survival [107]. To summarize, cell line-specific metabolic models were utilized for a wide variety of applications, including the identification of cancer vulnerabilities and synthetic lethal targets.

## Advantages and limitations of 13C-MFA and COBRA

Isotope tracing is widely used to probe intracellular metabolic activities in cancer cells. However, most studies still rely on manual assessment of measured metabolite isotopic labeling to qualitatively infer metabolic activities [8], while 13C-MFA is typically performed in a small number of labs that have expertise in these approaches. Manual inspection of isotopic labeling measurements is highly complicated and may bias the assessment of metabolic activities. For example, an increase in the fractional labeling of a metabolite under isotopic steady state may be falsely interpreted as an increase in flux through a producing pathway, although this may merely result from a change in the labeling of an upstream metabolic intermediate. With kinetic isotopic labeling measurements, faster labeling kinetics of a metabolite may be interpreted as increased flux, though this may result from a drop in the concentration of the metabolite. Isotope exchange effects also complicate manual interpretation of metabolic activities, with reactions close to chemical equilibrium simultaneously carrying flux in opposite directions [108, 109]. A comprehensive and quantitative view of metabolic fluxes derived by 13C-MFA enables us to evaluate how well we understand the working of complex metabolic systems and leads to important discoveries. For example, quantitative flux analysis of NADPH metabolism revealed that a major fraction of NADPH turnover is not explainable by the canonical NADPH-producing pathways, leading to the finding of a major contribution of folic acid metabolism to NADPH production [80]. Another example is with quantitative modeling of flux in cancer cells during anchorage-independent growth, showing that measured isotope labeling patterns of metabolites cannot be explained without taking into account subcellular compartmentalization effects, revealing citrate shuttling from the cytosol to mitochondria [55].

While both 13C-MFA and COBRA were demonstrated to be highly useful in cancer metabolic research, there are inherent limitations and complications with each approach. We provide a brief comparison of the two modeling approaches in terms of scope, required experimental data, and possible output (Table 1).

**Table 1:** A comparison between 13C-MFA and COBRA

	13C-MFA	COBRA
Network size	Small-scale (typically central metabolism) Difficult to determine network model boundaries Experimentally and computationally hard to extend for larger networks	Genome-scale Enables finding activity of non-canonical metabolic pathways Potential false prediction of non-canonical metabolic activities due to the inclusion of reactions with weak biochemical evidence in the network model
Typical experimental inputs	Biomass composition, growth rate, and metabolite uptake and secretion rates	
Computational requirements	Isotope tracing measurements; potentially absolute metabolite concentrations	A variety of ‘omics’ datasets Requires simplifying assumptions for integrative analysis
	Mostly hard non-convex optimization problems solved heuristically	Mostly computationally tractable optimizations (linear or quadratic programming)
Determining a unique flux solution	Typically possible Assessing uncertainty with confidence intervals	Requires simplifying optimizations (e.g., maximal growth rate)
Compartmentalization	Partially addressed with specific tracers, compartment-specific markers, cell fractionation	Addressed via simplifying optimization assumptions
Applicability	Inferring fluxes in a specific condition	
	–	Predict flux adaptation following chemical/genetic alterations

In terms of the scope of metabolic systems analyzed, COBRA is typically applied to infer flux via genome-scale metabolic networks, while 13C-MFA is applied to inspect central metabolism (typically spanning glycolysis, TCA cycle, and the pentose phosphate pathway). Analyzing genome-scale metabolic networks enables COBRA to reveal non-canonical pathways with an important contribution to some cancer cells. However, it can falsely predict flux through enzymatic reactions that were included in the model based on weak biochemical evidence. Further work by the metabolic modeling community is needed to further refine and extend the existing genome-scale metabolic network reconstructions based on accumulating knowledge of enzymatic activities in human cells. An important future challenge for COBRA methods is improving the reliability of biochemical enzymatic activities that are included in the model. With 13C-MFA, on the other hand, it is challenging to determine the boundaries of the analyzed metabolic system, while reactions that are left out of the model could potentially bias flux estimation. Applying 13C-MFA for larger scale networks is an experimentally challenging task which requires the measurement of metabolite isotopic labeling outside the central metabolism. Furthermore, it is highly computationally challenging to apply 13C-MFA for genome-scale networks, though some attempts in this direction have been made [110, 111]. Further work is required to make such genome-scale 13C-MFA methods more accessible for the research community.

While both 13C-MFA and COBRA rely on measurements of metabolite uptake and secretion rates for flux estimation, 13C-MFA that relies on isotope tracing measurements is more experimentally demanding. Omics data, and specifically genomics, transcriptomics, proteomics, and metabolomics, can be utilized as input by COBRA methods, though this typically relies on simplified heuristics that do not account for the complexity of regulatory and enzyme kinetic mechanisms. A major open challenge in COBRA is developing improved methods for utilizing quantitative proteomics and metabolomics data for flux inference via enzyme-mechanistic models accounting for kinetic and thermodynamic considerations.

In terms of the ability to uniquely infer flux, this is typically possible with 13C-MFA applied to analyze flux in central metabolism, rigorously evaluating flux confidence intervals. With COBRA, over-simplified optimality assumptions are typically employed to derive unique fluxes (e.g., parsimonious FBA [75]). Subcellular compartmentalization is typically accounted for in genome-scale metabolic network models analyzed by COBRA (though the prediction of flux by mitochondrial versus cytosolic enzymes is based on simplifying optimization criteria rather than concrete measurements). With 13C-MFA, inferring subcellular flux is technically challenging and typically not accounted for. While several approaches have recently been proposed to infer compartmentalized fluxes via specific isotopic tracers or rapid cell fractionation, this remains as a major challenge.

In terms of common applications, both COBRA and 13C-MFA enable the inference of flux in cells based on measurements performed under a specific genetic and cell culture condition. Derived flux maps by these approaches provide a holistic understanding of metabolic processes, while changes in flux due to genetic or environmental perturbations provide means to examine metabolic regulation. The identification of induced flux through specific enzymes in cancer cells reveals the increased dependence on metabolic transformations that could be therapeutically targeted. Note that unlike 13C-MFA, COBRA can further address the more challenging task of predicting how metabolic flux will be rewired in response to genetic or pharmacological interventions in silico, providing means to investigate potential anti-cancer drug targets.

## Concluding remarks

Overall, COBRA and 13C-MFA provide complementary capabilities for understanding the rewiring of metabolic flux in cancer. While 13C-MFA analyzes isotopic tracing measurements to provide an accurate quantitative view of flux through central metabolic pathways, COBRA analyzes flux through genome-scale metabolic networks based on physicochemical constraints and ‘omics’ data integration. In some cases, isotope tracing is used to quantify specific fluxes in human tissues under different physiological conditions, while these are used as inputs for COBRA-based flux analysis on a genome scale [112, 113]. In others, COBRA flux predictions are validated by comparison with 13C-MFA inferred fluxes [80]. Given the ever-growing interest in probing cellular metabolic fluxes, we expect COBRA and 13C-MFA to continue playing an important role in cancer metabolic research.

## Data Availability

Not applicable.
